# Personal

**Published:** 1902-01

**Authors:** 


### PERSONAL.

DR. NELSON H. HENRY, of New York, who has represented the 5th District in the Assembly for the past three years has been appointed by Governor Odell to be Adjutant General of the National Guard. His connection with the National Guard dates from 1883. He is now chief surgeon on the staff of Gen. Roe with the rank of colonel. He served as a division surgeon during the Spanish War in the camps of Tampa, Fla., and Huntsville, Ala. Dr. Henry, who now assumes the title of general, is an able officer and his appointment is received with favor by the guard.

DR. HARVEY R. GAYLORD, of Buffalo, has accepted an invitation to become one of the associate editors of the JOURNAL. Dr. Gaylord's accomplishments are well known. He is professor of surgical pathology at the University of Buffalo, pathologist at the State Laboratory, and attending surgeon at the Erie County Hospital. The JOURNAL regards his addition to the staff with great satisfaction, and we feel sure its readers will look upon it with equal favor.

DR. B. H. DAGGETT, of Buffalo, contributes to the current number of the Girard (Pa.) *Cosmopolite*, an article on the Pan-

American police force. As chief examiner of applicants for appointment to police duty at the Exposition, Dr. Daggett had an experience full of striking incidents, some of which were amusing. There were 4,700 applicants from all parts of the United States. Of these 1,278 appeared and were examined, 350 of whom passed to the eligible list. The article is one of interest.

Dr. John W. Riley, U. of B., '01, who has been house surgeon of the Emergency Hospital for the past eight months, resigned to take effect January 1, 1902. He will open an office at Eagle and Cedar Streets, for the practice of his profession. He was at the Fitch Hospital for two years before graduation and has had much surgical experience.

Dr. Robert E. Doran, formerly of Buffalo, and now a member of the staff of the Willard State Hospital, has been appointed first assistant physician at the Craig Colony for epileptics, Sonyea, N.Y.

Dr. Nelson W. Wilson, of Buffalo, has removed his office to 49 Niagara Street. His residence remains at 153 Seventh Street. Hours: 10-12 and 4-6. Telephone, Seneca, 76.

Dr. W. J. O'Donnell, of Buffalo, has received an appointment to the staff of St. Mary's Maternity Hospital on Edward Street. His duties there began December 1, 1901.

Dr. George F. Mills, of Buffalo, has been appointed junior physician of the Manhattan State Hospital.

